# Investigation into the cellular origins of posterior regeneration in the annelid *Capitella teleta*

**DOI:** 10.1002/reg2.94

**Published:** 2017-12-06

**Authors:** Danielle M. de Jong, Elaine C. Seaver

**Affiliations:** ^1^ Whitney Laboratory for Marine Bioscience University of Florida St Augustine FL 32080 USA

**Keywords:** annelid, *Capitella*, cell migration, regeneration, stem cells

## Abstract

Many animals can regenerate, although there is great diversity in regenerative capabilities. A major question in regenerative biology is determining the cellular source of newly formed tissue. The polychaete annelid, *Capitella teleta*, can regenerate posterior segments following transverse amputation. However, the source, behavior and molecular characteristics of the cells that form new tissue during regeneration are largely unknown. Using an indirect cell tracking method involving 5′‐ethynyl‐2′‐deoxyuridine (EdU) incorporation, we show that cell migration occurs during *C. teleta* posterior regeneration. Expression of the multipotency/germ line marker *CapI‐vasa* led us to hypothesize that stem cells originate from a multipotent progenitor cell (MPC) cluster, migrate through the coelomic cavity, and contribute to regeneration of tissue. We show that the capacity for posterior regeneration and segment formation is greater with than without the MPC cluster. Finally, we propose a working model of posterior regeneration in *C. teleta*. This work is the first in *C. teleta* that addresses the potential source of cells contributing to posterior regeneration, and may provide clues as to why some animals are highly successful regenerators.

## 1 INTRODUCTION

Many animals can regenerate, although there is great diversity in relative regenerative capabilities. For example, some animals are able to regenerate their complete body from only a few thousand cells (e.g., some planarians), while others can regenerate only a single cell or tissue type (e.g., cardiac muscle in zebrafish) (Bely & Nyberg, 2010). One of the most intriguing questions in regeneration biology is the cellular source of new tissue that is formed (see Tanaka & Reddien, 2011). Cells involved in regeneration of new tissue are assumed to be derived from one or more of a number of different sources: by division of stem cells, by dedifferentiation and subsequent division of undifferentiated cells, or via transdifferentiation of pre‐existing cell types. In addition, migration of multiple cell types to the wound site has been reported in a diverse number of animals, such as cnidarians, planarians, zebrafish, and axolotls (Bradshaw, Thompson, & Frank, 2015; McCusker, Bryant, & Gardiner, 2015; Reddien & Alvarado, 2004; Tahara, Brush, & Kawakami, 2016).

Annelids, the segmented worms, have long been models for regeneration studies, due to their impressive regeneration abilities and the wide range of regenerative abilities in this phylum (see Bely, 2006; Berrill, 1952). There has been an ongoing debate regarding the cellular source of the regenerated tissue in annelids, and most studies have been based on examination of fixed tissue (see Bely, 2014), with a recent exception of a live imaging study in the annelid *Pristina leidyi* (Zattara, Turlington, & Bely, 2016). From these studies, it appears that many of the cells that contribute to the blastema arise locally from the area of the wound site. It is thought that such cells dedifferentiate, proliferate, and then redifferentiate, and such a scenario is thought to occur for regeneration of tissues such as the gut, the outer epidermal epithelium, and muscle. In contrast, there is also evidence for migration of stem cells from a distant site. In response to amputation, multiple cell types migrate towards the wound site. Some of these migrating cells are thought to mediate an immune response, although one population known as neoblasts (Randolph, 1891, 1892), have been classified as a putative stem cell population. To date, cells that closely fit the description of neoblasts have been described mostly in clitellates, but also in some polychaetes (Bilello & Potswald, 1974; Cornec, Cresp, Delye, Hoarau, & Reynaud, 1987; Faulkner, 1929, 1932; Krecker, 1923; Probst, 1931; Randolph, 1891, 1892; Stephan‐Dubois, 1954; Stolte, 1929; Sugio et al., 2008; Tadokoro, Sugio, Kutsuna, Tochinai, & Takahashi, 2006; Zattara, Turlington, & Bely, 2016). Regeneration abilities are not limited to species with neoblasts; annelids that lack cells with obvious neoblast characteristics can regenerate (Herlant‐Meewis, 1964; Krecker, 1923; Myohara, 2012; Stone, 1933). The role of neoblasts in annelid regeneration has not been experimentally tested.

The polychaete annelid *Capitella teleta* displays robust posterior (but not anterior) regeneration following transverse amputation of body segments. Within 1 day of amputation, wound healing has occurred, and by 2 days a regeneration blastema is formed. The blastema is filled with actively proliferating cells and axonal extensions from the ventral nerve cord. During the following days, new tissue types appear, including ectodermal epithelia, gut, circular and longitudinal muscles, and neurons. By 12 days post‐amputation (dpa), there are typically between three and 13 newly formed segments (de Jong & Seaver, 2016). The source of cells that generate the blastema is currently unknown, be it via proliferation of stem cells, cell migration, dedifferentiation, or transdifferentiation. Determining the source of cells recruited to form new tissue during regeneration will allow us to gain a greater understanding of the regeneration process, such as how *C. teleta* can regenerate multiple tissue types.

In this study, we investigated the cellular source of regenerating tissue in *C. teleta*. We hypothesized that a source of cells distant to the wound site makes a contribution to the regenerate. To test whether there is cell migration during *C. teleta* posterior regeneration, we used an indirect method involving incorporation of 5′‐ethynyl‐2′‐deoxyuridine (EdU) and 5′‐bromo‐2′‐deoxyuridine (BrdU) to label and track cells in living animals. We used the expression of *CapI‐vasa* to mark putative stem cells present in the coelomic cavity, and analyzed their morphology and spatial distribution following transverse amputation. *CapI‐vasa* is also expressed by a second population of cells that was previously identified as a putative primordial germ cell (PGC) cluster (Dill & Seaver, 2008). Whether the putative PGC cluster has a restricted role in formation of the germ line is not known. Expression of the orthologs of *vasa*, *nanos* and *piwi* are not restricted to the PGC cluster, but are also expressed in somatic stem cells of the posterior growth zone in larvae, juveniles, and adults in *C. teleta* (this study; Dill & Seaver, 2008), as well as in tissues outside the germ line in other annelids including *Platynereis*, *Enchytraeus*, and *Tubifex* (Oyama & Shimizu, 2007; Ozpolat & Bely, 2016; Rebscher, Zelada‐González, Banisch, Raible, & Arendt, 2007; Sugio et al., 2008). Therefore, we investigated the possibility that the putative PGC cluster is actually a heterogeneous population of multipotent stem cells, and serves as a source of somatic stem cells during posterior regeneration. Henceforth we refer to the previously named PGC cluster as the multipotent progenitor cell (MPC) cluster. We assessed the relative capacity for posterior regeneration in juveniles with and without the putative MPC cluster, using axon outgrowth, cell proliferation, and number of regenerated segments as markers of regenerative capability.

## 2 RESULTS

### 2.1 Proliferating cells migrate into the blastema during posterior regeneration

**Figure 1:**
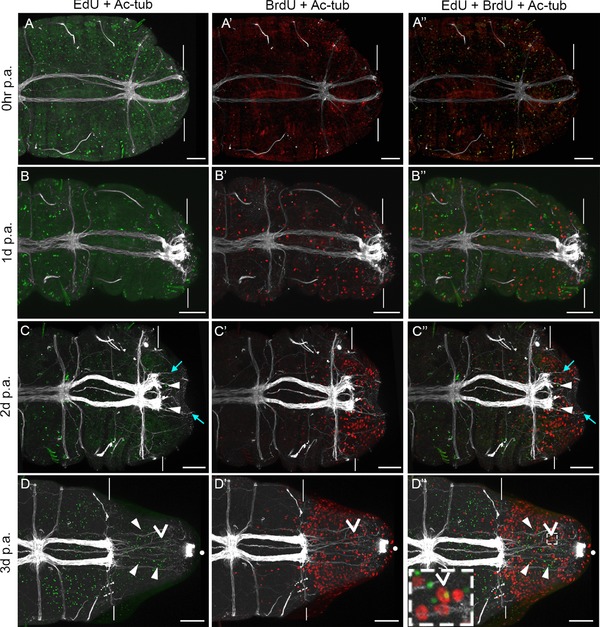
Migration of cells during posterior regeneration. All panels show confocal stacks of posterior ends of amputated juveniles in ventral view, with anterior to the left. The panels in each row are from a single individual. Amputations were conducted at the boundary of segments 10 and 11. White vertical lines indicate the approximate position of amputation, and all tissue to the right of the lines is regenerated tissue. The time following amputation is listed to the left of the rows, and the antibody/chemical represented in each panel is listed at the top of the columns. (A)−(D) EdU incorporation and anti‐acetylated α‐tubulin reactivity; (A′)−(D′) BrdU incorporation and anti‐acetylated α‐tubulin reactivity; (A″)−(D″) EdU incorporation, BrdU incorporation, and anti‐acetylated α‐tubulin reactivity. Filled white arrowheads in (C), (C″), (D) and (D″) show examples of EdU‐positive cells within the regenerated tissue. Pale blue arrows in (C), (C″) show examples of axons projecting into regenerating tissue. The open arrowhead in (D′) and (D″) indicates a cell that is both EdU and BrdU positive. The inset in (D″) is a high magnification image of tissue outlined by the white dotted rectangle. White dots in (D)−(D″) show the position of cilia of the hindgut. Chaetae are autofluorescent and are visible in panels (A)−(A″), (B), (B″), (C)−(C″). The scale bar in all panels represents 50 μm

To investigate a possible distant source of cells that contribute to new tissue formed during regeneration of posterior segments, we performed a set of experiments we termed “EdU pulse−amputate−wait−BrdU pulse” (Fig. 1). In these experiments, uncut juveniles were incubated in EdU, which was incorporated into cells undergoing the S‐phase of cell cycle during a 1 h exposure time. We used this as an indirect method to label and track cells in living animals over time. Following EdU incorporation, juveniles were amputated and allowed to regenerate for up to 3 days. Immediately before fixation, juveniles were exposed to a second DNA synthesis marker, BrdU, for 1 h. The location of both EdU‐positive and BrdU‐positive cells was subsequently visualized. EdU‐positive nuclei in the regenerating tissue show cells that originated in pre‐existing segments and are now present in the new tissue. BrdU‐positive nuclei indicate cells that were actively dividing in the animal immediately before fixation. A nucleus labeled with both EdU and BrdU in regenerated tissue indicates a cell that was previously dividing in segments anterior to the wound that migrated to new tissue, and subsequently divided a second time. Ventral nerve cord architecture was also visualized by anti‐acetylated α‐tubulin reactivity, which allowed for monitoring changes in the nervous system during blastema formation. In addition, because amputation results in an abrupt termination of the nerves of the ventral nerve cord, this labeling provides precise determination of the amputation site.

EdU‐positive and BrdU‐positive cells are scattered throughout the body in animals fixed at 0−1 h post‐amputation (hpa), which is typical for actively growing 2‐week‐old juveniles (Fig. 1A‐A″). At 1 dpa, wound healing has occurred but a regeneration blastema has not formed, and specimens resemble individuals at 0−1 hpa (Fig. 1B‐B″). At 2 dpa, a small regeneration blastema has formed, and fine axonal projections extend into the new tissue (Fig. 1C, pale blue arrows). Several EdU‐positive cells are present in this new tissue, along with a multitude of actively dividing cells, evidenced by BrdU incorporation (Fig. 1C‐C″). At 3 dpa, a large regeneration blastema has formed, and it contains many EdU‐positive cells, indicating migration of cells into the blastema (Fig. 1D). In addition, the blastema contains a large number of actively dividing BrdU‐positive cells (Fig. 1D′). This pattern is typical for a regeneration blastema of this stage (de Jong & Seaver, 2016), where proliferating cells are detectable in all three germ layers of the forming blastema (Fig. S1). In three independent EdU pulse−amputate−wait−BrdU pulse experiments, almost all blastemas at 2 dpa and 3 dpa contain EdU‐positive cells (*n* = 40/42). In animals with EdU‐positive cells, the number in the blastema ranged from only a few cells (two to three) to many cells (over 50). In general, at 2 dpa most animals possessed fewer than 25 EdU‐positive cells in the blastema, while at 3 dpa most animals possessed more than 25 EdU‐positive cells in the blastema. In addition, we occasionally see cells in the regeneration blastema at 2 dpa and 3 dpa that are both EdU‐positive and BrdU‐positive (Fig. 1D″, open arrowhead).

**Figure 2:**
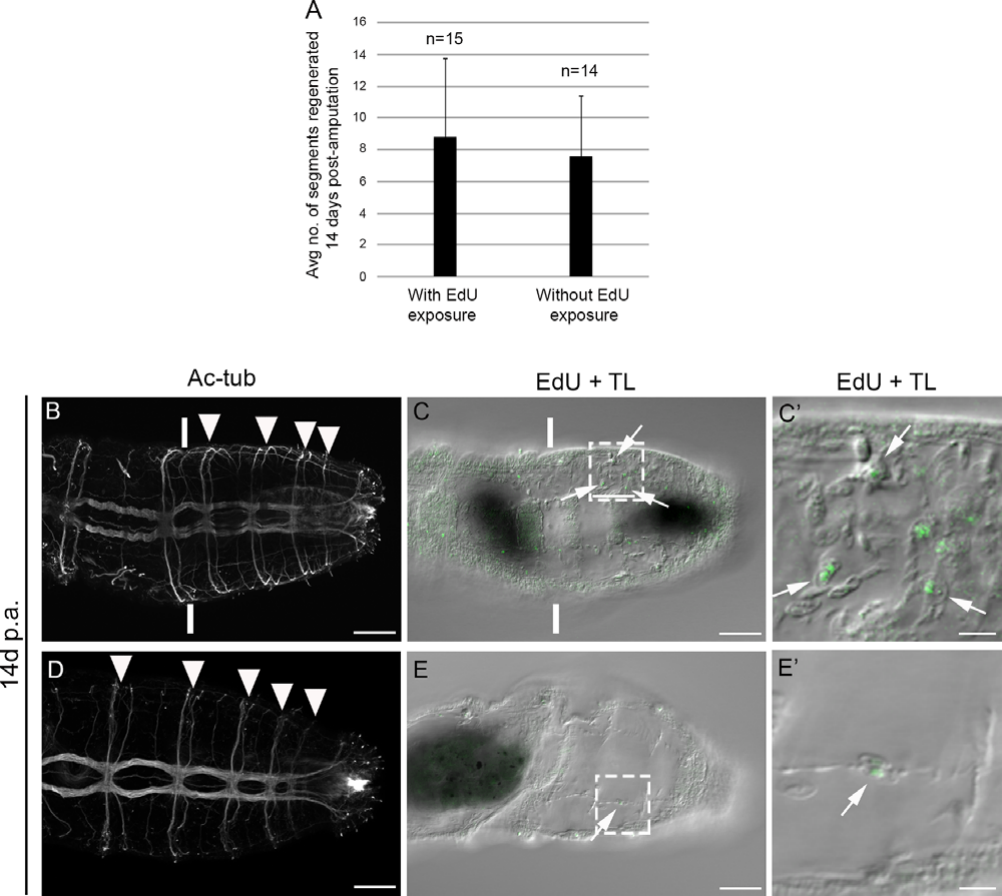
Robust regeneration of posterior segments following EdU exposure. (A) The average number of segments regenerated 14 days following amputation in animals either exposed to EdU or not exposed to EdU before amputation. Numbers at the top of the columns indicate the number of juveniles sampled. Error bars show the standard deviation from the mean. (B)−(E′) Confocal stacks of posterior ends of two different amputated juvenile individuals in ventral view, with anterior to the left. The panels in each row are from a single individual. Each individual was exposed to EdU for 1 h, amputated at the boundary of segments 10 and 11, and was then allowed to regenerate for 14 dpa. Vertical white lines in (B) and (C) indicate the approximate position of amputation. The amputation site in (D) and (E) is anterior to the field of view. (B), (D) Anti‐acetylated α‐tubulin reactivity that labels neurites. Filled white arrowheads in (B) and (D) show regenerated segments. (C), (C′), (E), (E′) EdU incorporation (green) combined with a transmitted light image of the regenerated tissue (EdU + TL). High magnification images in (C′) and (E′) correspond to the area defined by dotted rectangles in (C) and (E) respectively. White arrows in (C)−(C′) and (E)−(E′) indicate EdU‐positive cells within the regenerated tissue. Ac‐tub, anti‐acetylated tubulin; TL, transmitted light. Scale bars in (B)−(C), (D)−(E) represent 50 μm; scale bars in (C′), (E′) represent 10 μm

In order to ascertain if the initial incubation in EdU negatively affected subsequent posterior regeneration, we compared regeneration of animals that had been exposed to EdU with those that had not. One set of juveniles were amputated at the boundary of segments 10 and 11 following a 1 h EdU incubation. A second set of juveniles were treated similarly but were not exposed to EdU. Both sets of animals were allowed to regenerate for 14 days, and the number of segments that had regenerated were counted using the presence of segmentally repeated peripheral nerves through anti‐acetylated α‐tubulin labeling as a marker of mature ganglia (Fig. 2A). There is not a significant difference in the number of segments regenerated comparing animals that had been exposed to EdU and those that had not been exposed to EdU. In addition, EdU‐positive cells are visible in the new tissue, where they had become incorporated into new segments. This indicates that cells that had incorporated EdU 14 days earlier remain healthy, and contribute to differentiated tissues in the regenerated segments (Fig. 2B−E′). For example, EdU‐positive cells are clearly incorporated into differentiated mesodermal structures (Fig. 2C′, E′).

### 2.2 *CapI‐vasa*‐expressing cells are observed in the coelomic cavity of juveniles, and are most often located posterior to the MPC cluster

**Figure 3:**
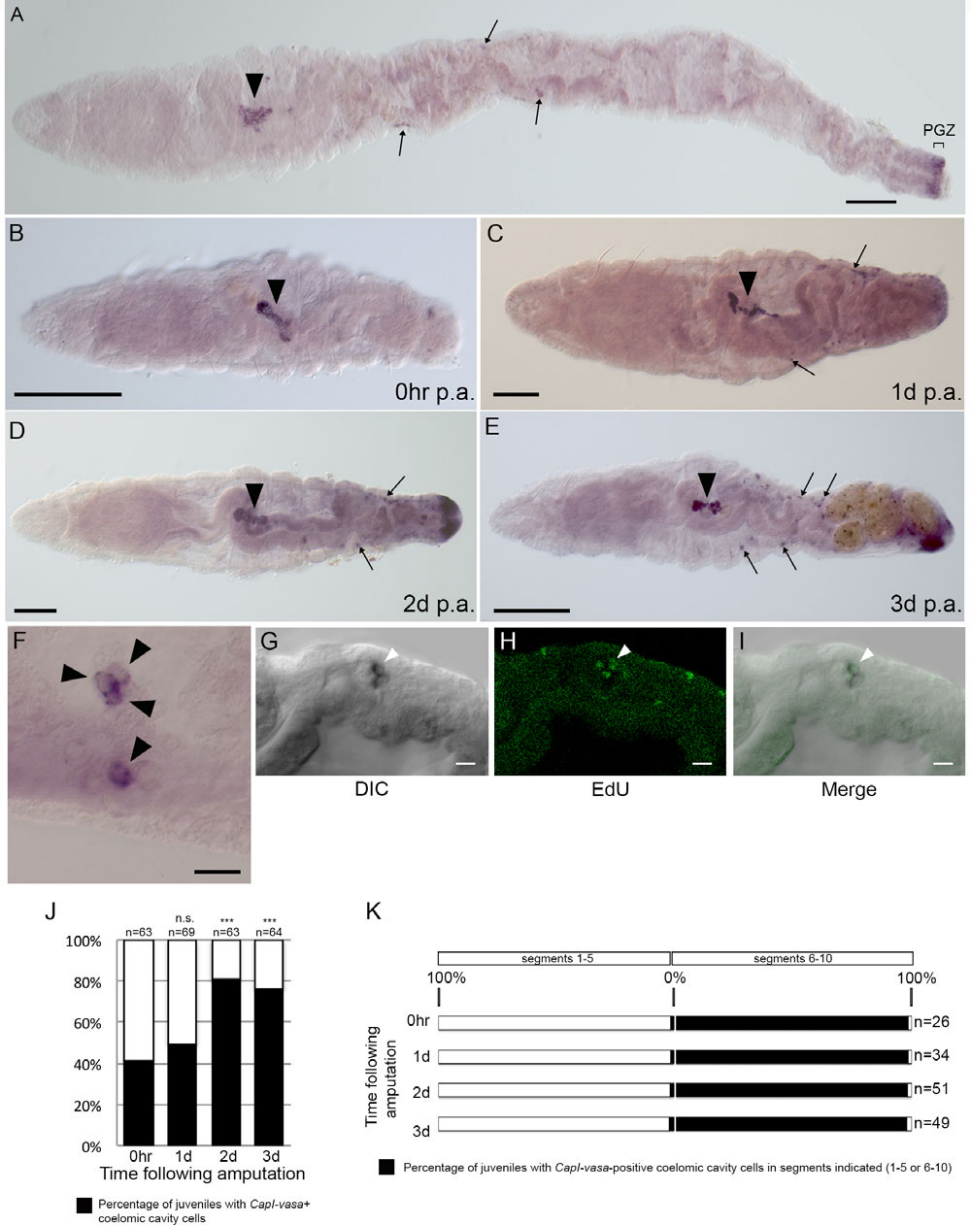
Characteristics of *CapI‐vasa*‐positive cells in the coelomic cavity during regeneration. (A)−(E) A ventral view, with anterior to the left. (F)−(I) A lateral view with anterior to the left. (A)−(E) Differential interference contrast (DIC) images of *CapI‐vasa*‐expression domains, in uncut juveniles (A), immediately after transverse amputation at segment 10 (B), or 1 dpa (C), 2 dpa (D), or 3 dpa (E). PGZ and the associated bracket show the location of the posterior growth zone in (A). Filled black arrowheads in (A)−(E) show the position of the multipotent progenitor cell (MPC) cluster which is consistently located in segment 5, and usually also extends to include part of adjacent segments 4 and/or 6. Black arrows show examples of *CapI‐vasa*‐positive cells in the coelomic cavity. (F) Black arrowheads pointing to a high magnification image of *CapI‐vasa*‐positive cells in the coelomic cavity. (G)−(I) Confocal images from a single individual generated from a subset of slices from a *z*‐stack, showing *CapI‐vasa* expression (G), EdU incorporation (H), and the overlap of the two (I). Filled white arrowheads in (G), (H), and (I) show an example of an EdU‐positive cell within the *CapI‐vasa* expression domain. (J) The proportion of juveniles with *CapI‐vasa*‐positive cells in the coelomic cavity immediately following amputation, or 1, 2, or 3 days following amputation. Numbers at the top of the columns indicate the number of juveniles sampled. Statistical significance compared with 0 h controls with a *P* value <0.01 is denoted by ***, while n.s. indicates that the difference in proportions was not statistically significant. (K) The axial distribution of *CapI‐vasa*‐positive cells in the coelomic cavity immediately following transverse amputation at segment 10 (0 hpa), or 1, 2, or 3 dpa. The percentage of juveniles with *CapI‐vasa*‐positive coelomic cavity cells in segments 1−5 or 6−10 is indicated with black bars. Numbers to the right of rows indicate the number of juveniles sampled at each time point. Scale bars in (A)−(E) represent 100 μm. Scale bars in (F)−(I) represent 10 μm

In uncut juveniles, *CapI‐vasa* is expressed in three domains (Fig. 3A): cells in the posterior growth zone, dispersed cells in the coelomic cavity (black arrows), and cells in the MPC cluster (filled black arrowhead). We monitored *CapI‐vasa* expression following amputation in cells in the coelomic cavity (see below) and in the MPC cluster (see the next section).

*CapI‐vasa*‐expressing cells are present within the coelomic cavity in a subset of both uncut and regenerating juveniles (Fig. 3A−E). These cells are approximately 10 μm in diameter, and occur as individual cells or in small clusters of two to four cells (Fig. 3F). In addition, the cells occasionally divide, as determined by EdU incorporation (Fig. 3G−I). Although it was technically impractical to count the number of *CapI‐vasa*‐expressing cells within the coelomic cavity due to their small size and three‐dimensional arrangement within the body cavity, the percentage of juveniles that contained *CapI‐vasa*‐expressing coelomic cavity cells was calculated (Fig. 3J). One set of animals was fixed immediately following amputation between segments 10 and 11 (0 hpa), and in these animals 41% of individuals have *CapI‐vasa*‐expressing cells within their coelomic cavity (*n* = 26/63). A similar proportion is observed at 1 dpa (49%, *n* = 34/69). In contrast, there was a substantial increase in the proportion of juveniles containing *CapI‐vasa*‐expressing cells in the coelomic cavity at 2 dpa (81%, *n* = 51/63) and at 3 dpa (77%, *n* = 49/64). The proportion of juveniles that contain *CapI‐vasa*‐expressing cells in their coelomic cavities at 2 dpa and 3 dpa are significantly different from the proportion containing *CapI‐vasa*‐expressing cells at 0 hpa (*P *< 0.01 in both cases).

The *CapI‐vasa‐*expressing cells do not appear to have a specific location within each segment, other than being present in the coelomic cavity. However, when their relative axial position is taken into account, it is clear that these cells are restricted to particular segments. For each individual, the segment number where *CapI‐vasa*‐expressing cells was seen at four time points following amputation was recorded (0 hpa, 1 dpa, 2 dpa and 3 dpa). At all time points, the vast majority of individuals (96%, *n* = 25/26 at 0 hpa; 97%, *n* = 33/34 at 1 dpa; 98%, *n* = 57/58 at 2 dpa; and 98%, *n* = 48/49 at 3 dpa) contain these cells in segments 6−10, while only a small fraction of the cells are located in segments 1−5 (Fig. 3K; 4% at 0 hpa, 3% at 1 dpa, 2% at 2 dpa and 2% at 3 dpa). When present, *CapI‐vasa*‐expressing cells are most often present in more than one segment (Fig. S2). In cases where they were observed in only one segment, the number ranged from only a few (approximately three to six cells) to many (too numerous to count). The MPC cluster is consistently located in segment 5 of juveniles, and also usually extends to include part of adjacent segments 4 and/or 6 (see below). Thus, almost all *CapI‐vasa*‐expressing coelomic cavity cells are positioned between the MPC and the wound site, which led us to hypothesize that cells of the MPC cluster migrate through the coelomic cavity and contribute to the blastema during posterior regeneration.

### 2.3 Morphological characteristics of the MPC cluster and its response to transverse amputation and posterior regeneration

Previous studies in *C. teleta* identified a cluster of cells in larvae, juveniles, and adults that express the germ and stem cell markers *vasa*, *nanos*, and *piwi* (Dill & Seaver, 2008). This group of cells is localized in the coelomic cavity and suspended by mesentaries, sandwiched between the ventral nerve cord and gut, and has been hypothesized to play a role in formation of the germ line. Although *vasa*, *nanos*, and *piwi* show indistinguishable expression patterns in the MPC cluster, expression analysis of a fourth marker, *Ct‐myc*, indicates molecular heterogeneity within the MPC cluster (see below). In addition, *vasa*, *nanos*, and *piwi* are all also expressed in undifferentiated cells of the posterior growth zone. Therefore, we now consider the possibility that this cluster may be a heterogeneous population of multipotent stem cells that can contribute to somatic tissues during regeneration, in addition to formation (or replacement) of the germ line.

**Figure 4:**
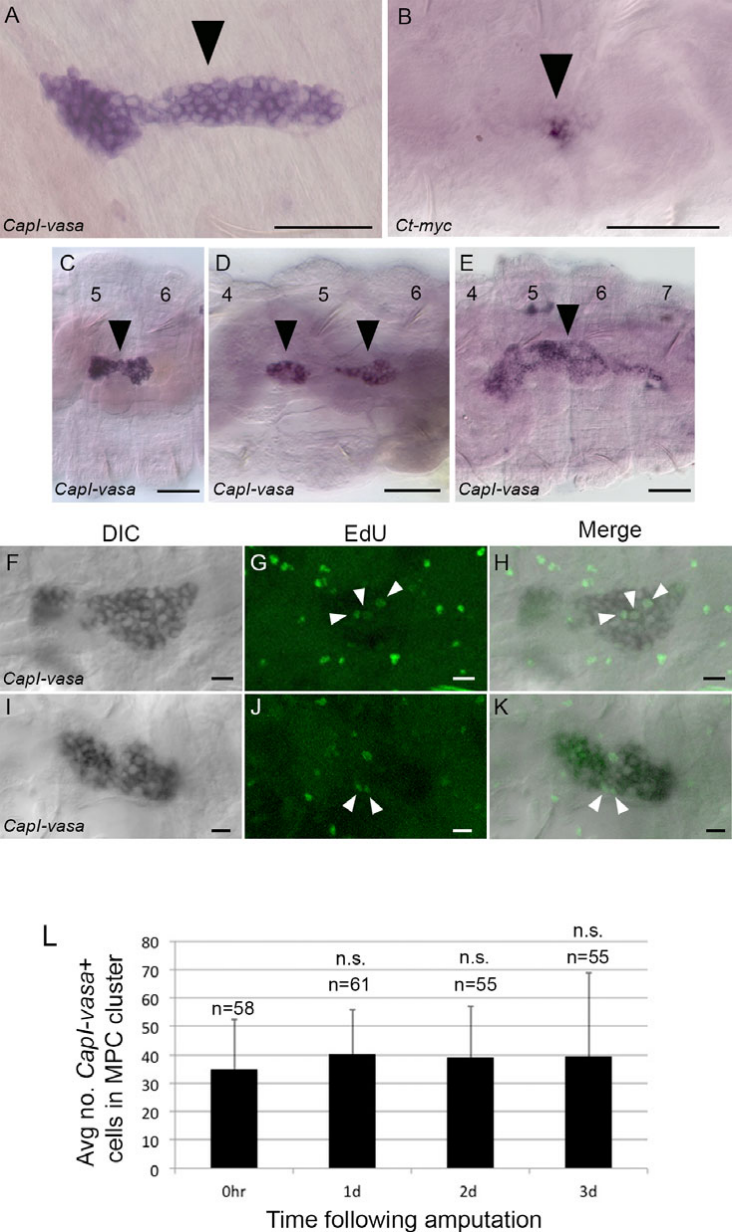
Cellular and morphological characteristics of the multipotent progenitor cell (MPC) cluster in *C. teleta*. All panels show a ventral view, with anterior to the left. (A), (C), (D), (E) DIC images of the *CapI‐vasa* expression domain(s); (B) DIC image of the *Ct‐myc* expression domain. (F)−(K) Confocal images generated from a subset of slices from a *z*‐stack, showing *CapI‐vasa* expression and EdU incorporation. (F)−(H) are from a single individual, as are (I)−(K). (A)−(E) A magnified image of the MPC cluster, indicated by the filled black arrowheads. Numbers in (C)−(E) indicate segment numbers where expression is seen. The MPC cluster is either a dumbbell‐shaped group of cells (C), two separate groups of cells (D), or a single elongated group of cells (E). Filled white arrowheads in (G), (H) and (J), (K) show EdU‐positive cells within the *CapI‐vasa* expression domain. (L) The average number of *CapI‐vasa*‐positive cells within the MPC cluster immediately following transverse amputation (0 hpa), and 1, 2, or 3 dpa. Error bars show the standard deviation from the mean. Numbers at the top of the columns indicate the number of individuals sampled, and n.s. indicates that cell numbers are not significantly different at 1−3 dpa, compared with 0 hpa controls. Scale bars in (A)−(E) represent 50 μm. Scale bars in (F)−(K) represent 10 μm

To further characterize the MPC cluster, the axial position, morphology, cell division characteristics, and response to transverse amputation of the MPC in 2‐week post‐metamorphic juveniles were investigated. We used *CapI‐vasa* as a marker of the putative MPC cluster (Fig. 4A, black arrowhead). The total length of the MPC cluster along the anterior−posterior axis is approximately 135−140 μm, as defined by *CapI‐vasa* expression. The localization of the *CapI‐vasa* transcript to the cytoplasm and exclusion from the nucleus facilitate visualization of individual cells and their morphology. Each cell in the cluster is approximately 10 μm in diameter, with a large nuclear:cytoplasmic ratio (Fig. 4A). There are indications that the MPC cluster is molecularly heterogeneous. For example, expression of *Ct‐myc* is consistently localized to only a few cells within the MPC cluster (Fig. 4B). The MPC cluster has a consistent axial position during both growth and regeneration; it is located in segment 5 of juveniles, and also usually extends to include part of adjacent segments 4 and/or 6 (Figs. 4C−E and S2). The MPC cluster is typically elongated, although it can take on several morphological configurations in different individuals, including a simple dumbbell shape (Fig. 4C), two separate clusters of cells (Fig. 4D), or a single elongated group of cells (Fig. 4E). Occasionally, a subset of cells of the MPC cluster incorporates EdU during a 1 h exposure, indicating that these cells sometimes divide (Fig. 4F−K).

To investigate whether the number of cells within the MPC cluster changes in response to transverse amputation and subsequent regeneration, the number of *CapI‐vasa*‐positive cells in the MPC cluster was manually counted at different time points following amputation. Juveniles were amputated at the boundary between segments 10 and 11, and either fixed immediately (0 h), or allowed to regenerate for 1, 2, or 3 days. Subsequently, in situ hybridization was performed using *CapI‐vasa* as a probe, and the number of cells making up the putative MPC cluster was counted. The 0 h time point was representative of the number of cells in the cluster in 2‐week post‐metamorphic, unamputated juveniles. The number of cells within the MPC cluster does not significantly change during the first 3 days of regeneration compared to the 0 hpa time point (Fig. 4L; 1 dpa, *P *= 0.079; 2 dpa, *P *= 0.199; 3 dpa, *P *= 0.332).

### 2.4 Posterior regeneration in the presence and absence of the MPC cluster

**Figure 5:**
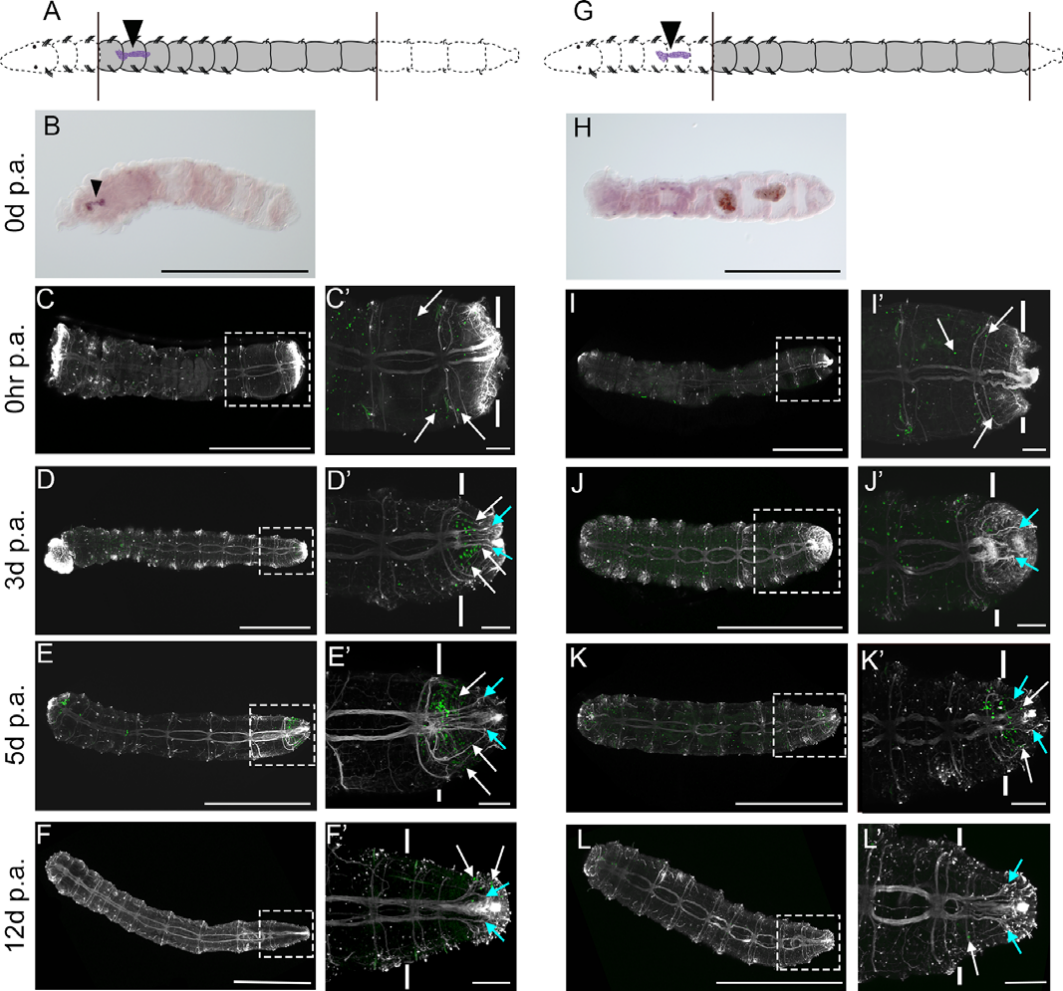
Comparison of regeneration in juveniles with and without the multipotent progenitor cell (MPC) cluster. All panels show images of juveniles in ventral view, with anterior to the left. The left schematic at the top of (A) shows a representation of the experimental manipulation for animals in (B)−(F′), and the right schematic at the top of (G) shows the experimental manipulation for animals in (H)−(L′). Black arrowheads in the schematics show the position of the MPC cluster, black lines show the position of amputation, and grey shading indicates the portion of animal that was retained after cutting. (B), (H) DIC images of *CapI‐vasa* expression. (C)−(F′), (I)−(L′) Confocal stacks of EdU incorporation in proliferating cells (green), and neurites labeled with anti‐acetylated α‐tubulin (grey). The time following amputation is listed to the left of each row. (C′)−(F′) and (I′)−(L′) are high magnification images of areas denoted by dotted rectangles in (C)−(F) and (I)−(L), respectively. Solid white lines in (C′)−(F′) and (I′)−(L′) indicate the approximate position of the posterior amputation site. White arrows in (C′) and (I′) show examples of EdU‐positive cells in pre‐existing segments, while white arrows in (D′)−(F)′ and (K′)−(L′) show examples of EdU‐positive cells in regenerating tissue. Pale blue arrows in (D′)−(F′) and (J′)−(L′) show examples of acetylated α‐tubulin axons projecting into regenerating tissue. Scale bars in (B)−(F) and (H)−(L) represent 500 μm. Scale bars in (C′)−(F′) and (I′)−(L′) represent 50 μm

To determine whether the MPC cluster is involved in posterior regeneration, we performed amputations that removed the MPC cluster. In a first set of experiments, a single amputation was made either anterior to the MPC cluster (between segments 3 and 4) or posterior to the MPC cluster (between segments 6 and 7). However, amputation anterior to the MPC cluster resulted in a high rate of mortality of animals (de Jong and Seaver, unpublished observations). Therefore, in a second set of experiments, two simultaneous anterior and posterior amputations were made. For the first group of animals, one transverse amputation was conducted between the third and fourth segments, and a second amputation between the thirteenth and fourteenth segments. In this group, the MPC cluster, which resides in segment 5, was retained (Fig. 5A). A second group of animals were amputated between both the sixth and seventh segments and the sixteenth and seventeenth segments, thereby removing the MPC cluster (Fig. 5G). Thus, while both groups had several anterior and posterior segments removed, all animals had 10 body segments remaining, and animals survived for the duration of the experiment. Following amputations, juveniles were either immediately fixed (0 hpa; Fig. 5B−C′, H−I′), or left to regenerate for 3 days (Fig. 5D, D′, J, J′), 5 days (Fig. 5E, E′, K, K′), or 12 days (Fig. 5F, F′, L, L′). Juveniles were then analyzed for cell proliferation (EdU incorporation), nerve extension (anti‐acetylated α‐tubulin reactivity), and formation of new segments (nuclear staining) in the posterior end of the regenerates.

**Figure 6:**
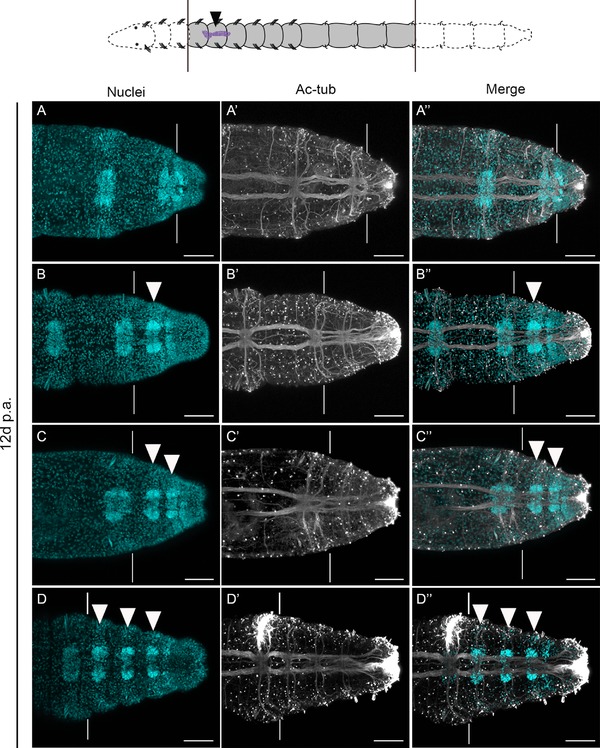
Regeneration of posterior segments in the absence of anterior segments. All panels show confocal stacks of posterior ends of amputated juveniles in ventral view, with anterior to the left. The schematic at the top shows a representation of the amputation scheme leading to the animals in (A)−(D″). The filled black arrowhead in the schematic shows the position of the MPC cluster, black lines show the position of amputation, and grey shading indicates the portion of animal that was retained after cutting. All panels show animals that were left to regenerate for 12 days before sampling. The panels in each row are from a single individual. White vertical lines in panels (A)−(D″) indicate the approximate position of the posterior amputation site. The specific stain or antibody is indicated at the top of the columns. (A)−(D) Hoechst 33342 staining labeling nuclei; (A′)−(D′) anti‐acetylated α‐tubulin reactivity labeling neurites; (A″)−(D″) a merge of Hoechst 33342 staining (blue) and anti‐acetylated α‐tubulin reactivity (white). Filled white arrowheads in (B), (B″), (C), (C″), (D) and (D″) point to newly formed ganglia in regenerated tissue. Scale bars represent 50 μm

At 0 hpa, animals both with and without the MPC cluster show EdU‐positive cells scattered throughout the body (Fig. 5C, C′, I, I′), which is typical for 2‐week post‐metamorphic, actively growing juveniles. As expected, amputation causes abrupt termination of the axons of the ventral nerve cord at both the anterior and posterior ends (Fig. 5C, C′, I, I′). By 3 dpa, approximately 35% (*n* = 6/17) of individuals with the MPC cluster have a blastema containing both EdU‐positive cells and axons that extend into the new tissue, while 41% (*n* = 7/17) of animals have axonal projections in the new tissue but do not have an accumulation of EdU‐positive cells in the new tissue (Fig. 5D, D′). In contrast, in animals without the MPC cluster, the proportion of individuals with a blastema containing EdU‐positive cells in addition to axons extending into the blastemal tissue is less than 7% (*n* = 1/15; Fig. 5J, J′). Most individuals without the MPC cluster have axonal projections into the new tissue but do not have an accumulation of EdU‐positive cells in the new tissue (87%, *n* = 13/15). In the group with the MPC cluster, 24% (*n* = 4/17) have neither axonal projections nor EdU‐positive cells in the blastema, in contrast to less than 7% (*n* = 1/15) in the group without the MPC cluster. At 5 dpa, all individuals with the MPC cluster (*n* = 10/10) have both axonal projections and EdU‐positive cells within the blastema (Fig. 5E, E′), compared to 89% of individuals without the MPC cluster (*n* = 8/9; Fig. 5K, K′). A single individual without the MPC cluster at 5 dpa has axonal projections but does not have an accumulation of EdU‐positive cells in the blastema (*n* = 1/9). At 12 dpa, all individuals (*n* = 14/14) with the MPC cluster present have axonal projections into the new tissue, in contrast to 78% of individuals without the MPC cluster (*n* = 7/9). No animals at this time point in either group have a detectable posterior growth zone, an area anterior to the pygidium containing a dense population of EdU‐positive cells (de Jong & Seaver, 2016), although scattered EdU‐positive cells can be seen in the new tissue (Fig. 5F, F′, L, L′). At 12 dpa, individuals with an intact MPC cluster formed between none and three new segments; the majority of individuals formed between one and three segments (86%, *n* = 12/14), while two individuals formed no segments (14%, *n* = 2/14). New segments were defined by the presence of newly formed, mature ganglia and peripheral nerves (Fig. 6). No individuals without the MPC cluster form new segments after regeneration for 12 days, with the exception of one individual which formed one new segment (11%, *n* = 1/9). In summary, juveniles with and without the MPC cluster undergo wound healing, and later show signs of cell division and projection of axons into the blastema. However, with the exception of one individual, only the animals with the MPC cluster form new segments.

## 3 DISCUSSION

### 3.1 Evidence for contribution of migrating cells to posterior regeneration in the polychaete annelid *Capitella teleta*

In this study, we present the first evidence of cell migration during posterior regeneration in *C. teleta*. Using a technique we term “EdU pulse−amputate−wait−BrdU pulse” to track a subset of cells in living animals, we show that, following transverse amputation, proliferating cells from pre‐existing segments migrate into the regeneration blastema. These observations are striking considering that a short EdU pulse, which labels only a subset of dividing cells, allowed us to visualize cell migration into the blastema. Furthermore, a proportion of these cells divide after migration. The fact that these migratory cells are dividing and at least some can continue to divide suggests that they are undifferentiated stem cells. These migratory cells contribute to differentiated cells and tissues in the regenerate. Our data add to previous results indicating migration of cells into the wound site during regeneration of various annelid species. However, to our knowledge, our results are the first demonstration through analysis of living tissue for a cellular contribution from migrating cells in any polychaete.

The origin of cells that form the blastema has long been the subject of intense research and speculation in annelids (Bely, 2014; Berrill, 1952; Clark & Clark, 1962; Cornec et al., 1987; Cresp, 1964; Faulkner, 1929, 1932; Herlant‐Meewis, 1964; Hill, 1970; Krecker, 1923; Zattara & Bely, 2011). Cell migration during annelid regeneration has been documented in multiple species, and appears to be a general feature of annelids (see Bely, 2014). Migration can initiate minutes after wounding and persist for at least 24 h after injury (Cameron, 1932; Cornec et al., 1987; Huguet & Molinas, 1994; Zattara et al., 2016). Histological studies of fixed specimens during various stages of regeneration have identified multiple different cell types that migrate, including undifferentiated stem cells that contribute to the regenerating tissue, known as neoblasts. The term neoblast was first coined to describe a cell type with unique characteristics observed in fixed tissues of the regenerating oligochaete *Lumbriculus* (Randolph, 1891, 1892). In this original description, neoblasts were characterized as large cells with round or oval nuclei present in the mesoderm of adult individuals. They were observed in most segments, and were present laterally on each side of the ventral nerve cord, between the nerve cord and the ventral setae. During regeneration, neoblasts proliferated and contributed to the reformation of ventral mesodermal tissue. Subsequent studies have used the term neoblasts to describe stem cells from a range of annelids and expanded the definition to include cells with additional features (Bely, 2014; Bilello & Potswald, 1974; Cornec et al., 1987; Faulkner, 1932; Herlant‐Meewis, 1964; Hyman, 1940; Krecker, 1923; Myohara, 2012; Randolph, 1891, 1892; Tadokoro et al., 2006). Morphologically, they have been described as either spindle shaped or round cells with a large nuclear to cytoplasmic, that reside on the intersegmental septa on the dorsal surface of the ventral nerve cord within the coelomic cavity. In the oligochaete *Enchytraeus*, cells with these features express *vasa*, and appear to migrate to the wound site following injury (Sugio, Yoshida‐Noro, Ozawa, & Tochinai, 2012; Sugio et al., 2008). The cells we observe to migrate into the regenerating tissue in *C. teleta* have some characteristics of neoblasts. For example, the initial EdU exposure shows that they were actively dividing before migration began, and co‐labeling with BrdU in some of these cells shows that they continue to divide. We hypothesize that a proportion of these dividing, migrating cells also express *vasa*, and therefore have a large nuclear to cytoplasmic ratio. However, rather than definitively identify the migratory cell population in *C. teleta* as neoblasts, we instead acknowledge that migration is occurring during regeneration and that these cells have a stem cell character. While some of the proliferating cells that migrate into the blastema in *C. teleta* are later incorporated into new segments, there may also be unlabeled cells that migrate into the newly forming tissue. For example, EdU incorporation only labels cells that are undergoing DNA synthesis at the time of exposure, and thus the 1 h pulses used in these experiments will probably only label a subset of a larger population of actively dividing cells. In addition, it is also possible that non‐dividing cells (e.g., differentiated cell types) also migrate, as reported in previous studies (see Bely, 2014), including in a live imaging study in *Pristina leidyi* (Zattara et al., 2016). These non‐proliferating migrating cells could then undergo a dedifferentiation or transdifferentiation step when they reach their final destination, or be part of an early immune response to the wound.

Evidence from fixed tissue studies in multiple annelids suggests that, in general, the formation of each germ layer in the new tissue is derived from the corresponding germ layer in the pre‐existing tissue (Bely, 2014; Berrill, 1952; Boilly, 1967, 1968; Clark & Clark, 1962; Cornec et al., 1987; Cresp, 1964; Hill, 1970). That is, ectoderm derives from ectoderm, mesoderm from mesoderm, and endoderm from endoderm. For example, in the sabellid *Sabella melanostigma*, all three germ layers appear to be involved in forming the blastema; ectodermal cells originate from ectoderm, endodermal cells contribute directly to outgrowth of endodermal epithelia, and mesodermal tissues arise from mesodermal coleomic cavity cells and from fragmentation of pre‐existing muscle, which probably dedifferentiates before dividing and redifferentiating (Hill, 1970). Migration of differentiated endodermal cells and subsequent incorporation into regenerating digestive tissue has also been documented in the oligochaete *Lumbriculus* (Tweeten & Reiner, 2012). Although our experiments do not specifically address the cellular origins of the different germ layers in the regenerate, our EdU pulse−wait−BrdU pulse experiments show that mesodermal structures in the regenerate are formed, at least in part, by EdU‐positive cells in the coelomic cavity (mesodermal layer) that have migrated from segments anterior to the wound. In addition, proliferating cells are present in the blastema of *C. teleta* in all three germ layers within 3 dpa (Fig. S1). These proliferating cells are likely to be incorporated into newly differentiated tissue during regeneration, and may contribute to the regenerate from outgrowth of existing tissue. As additional cell types that contribute to the regenerated tissue are revealed, e.g., by time‐lapse imaging of fluorescently labeled cells in live animals, we will be better able to determine the origins of these cells, and their contributions to the formation of each germ layer in the new tissue.

Although our results show the migration of EdU‐positive cells into the blastema, it should be noted that we are not able to determine from where these cells originate. For example, it is possible that the cells originate in several segments anterior to the wound, and then migrate posteriorly across multiple segments following an amputation event. Alternatively, migration may occur on a small scale. For example, cells could originate within the segment closest to the wound site, and migrate only within this segment. It is also possible that cells originate directly adjacent to the wound, so that migration need only occur over very small distances. A previous study in *C. teleta* quantified the number of dividing cells following transverse amputation in the three segments closest to the wound site, and compared the number of these cells to the number in the corresponding segments in uncut controls (de Jong & Seaver, 2016). In the first 24 hpa, there is a decrease in the number of dividing cells in the three segments closest to the wound site. However, by 2 dpa, cell proliferation recovers to levels comparable to uncut controls, and then increases over the following days. The initial observed decrease in the number of dividing cells might suggest that the origin of the proliferating, migrating cells are within the three segments proximal to the wound site. The migration of these cells would deplete the reserves available in these segments, resulting in the observed decrease noted in the previous study. The recovery and then subsequent increase in proportion of dividing cells in these segments could be due to the birth of new cells within these segments, or continued migration from cells further anterior. A second explanation to account for this decrease in proliferating cells proximal to the amputation site is that long‐range signals originating from the wound cause a shutdown of cell division, as has been proposed in the annelid *Pristina leidyi* (Zattara & Bely, 2013).

### 3.2 Identification of candidate migratory stem cells

Examination of the *CapI‐vasa* expression pattern in juveniles allowed us to identify two candidate cell populations that may serve as sources of migratory stem cells during posterior regeneration. Specifically, one population is the MPC cluster, and we propose that it serves as a cellular source of a second population of *CapI‐vasa*‐positive*^ ^*cells in the coelomic cavity. This second population of cells migrates through the coelomic cavity to the site of injury, and contributes to regeneration of posterior tissue. Several observations support our hypothesis. First, cells in both populations have a similar morphology and express *CapI‐vasa*. Second, during the first 3 days of regeneration, the proportion of juveniles that contain *CapI‐vasa*‐positive cells in the coelomic cavity increases. Finally, coelomic cavity cells are almost always located between the MPC cluster and the site of amputation. Although cells occasionally divide within the MPC cluster, the number of *CapI‐vasa‐*positive cells within the cluster does not change following transverse amputation. This result is best explained by maintenance of the population of cells in the MPC cluster through a homeostatic mechanism. That is, a cell within the cluster may divide, and the daughter cell migrates from the cluster to the wound site, while the mother cell remains in its original position. Such a self‐renewing strategy is characteristic for stem cells in multiple species and contexts (see Shenghui, Nakada, & Morrison, 2009; Tanaka & Reddien, 2011).

At all time points examined (0 hpa, 1 dpa, 2 dpa and 3 dpa), a small proportion of juveniles do not have detectable *CapI‐vasa*‐expressing coelomic cavity cells. It is possible that the expression of *CapI‐vasa* is dynamic within these cells, and therefore fixation at a specific time point results in a subset of individuals that do not express *CapI‐vasa*. Alternatively, the absence of these cells might reflect variability in regeneration capability among individuals. For example, the number of segments regenerated after 14 days varies widely (Fig. 2A). The absence of *CapI‐vasa*‐expressing cells in the coelomic cavity may account for some of this individual variation; individuals that do not have *CapI‐vasa*‐expressing coelomic cavity cells regenerate fewer segments than those that do. We cannot distinguish between these possibilities directly, as we do not currently have a way to label these cells in living animals.

Molecular heterogeneity was identified within the MPC cluster. Specifically, *Ct‐myc* is expressed in a subset of the cells that express *CapI‐vasa* (and presumably *CapI‐piwi1*, *CapI‐piwi2*, and *CapI‐nanos*) in the MPC cluster of *C. teleta*. This molecular heterogeneity within the MPC cluster could indicate different fates among cells. One annelid example in which differences in stem cell fates are reflected by molecular differences comes from the oligochaete *Enchytraeus*. Here, germline stem cells and germ cells express *piwi* and both *vasa* paralogs (*vlg‐1* and *vlg‐2*), while neoblasts (somatic stem cells) express only *vlg‐2* (Sugio et al., 2008). Similarly, in the planarians *Dugesia* and *Schmidtea*, all neoblasts (note that planarian neoblasts are distinct from annelid neoblasts) express *piwi*, but a subpopulation destined to form the germline expresses both *piwi* and *nanos* (Handberg‐Thorsager & Saló, 2007; Sato et al., 2006; Shibata, Rouhana, & Agata, 2010). In *C. teleta*, there may be further subpopulations of cells in the MPC cluster, coelomic cavity, or posterior growth zone that express unique combinations of stem cell markers. Unique combinations of markers might reflect differing potency within these populations. As more molecular markers are identified, it will allow us to further investigate the molecular heterogeneity and potential differences in developmental and regenerative potency of these cells.

### 3.3 Assessing the contribution of the MPC cluster to posterior regeneration

As a functional test of the importance of the MPC cluster to posterior regeneration, we compared the regenerative capability between animals with and without an MPC cluster. Due to the design of these experiments, the regeneration response was not as robust as in animals undergoing posterior regeneration with anterior segments intact. With intact anterior ends, there is substantial cell division in the blastema by 3 dpa (de Jong & Seaver, 2016). However, in our experiments, most animals in both groups did not consistently form a proliferative regeneration blastema until 5 dpa, suggesting that there is a delay in the onset of proliferation in the blastema. All experimental animals with the MPC cluster and a majority without the MPC cluster possessed EdU‐positive cells in the blastema at 5 dpa. At 12 dpa, most animals with the MPC cluster had regenerated one to three segments (85%, *n* = 12/14, Fig. 6), while most animals without the MPC cluster had not regenerated any segments (89%, *n* = 8/9; a single individual regenerated one segment). This is in stark contrast to the situation in animals with intact anterior ends, which regenerate between three and 13 segments after 12 days. In addition, a new posterior growth zone forms, which is indistinguishable from the growth zone seen in intact, actively growing animals (de Jong & Seaver, 2016). In our experiments, we did not observe the formation of a posterior growth zone in any animal from either group, regardless of the number of segments formed. The lack of a posterior growth zone in these animals would then, in turn, probably limit the formation of new segments. The limited number of regenerated segments and the lack of a posterior growth zone at 12 dpa is probably due to the inability of the animals to feed. A previous study noted that starvation of both intact and regenerating animals results in decreased cell proliferation and gene expression in *C. teleta* (de Jong & Seaver, 2016). A direct link between cell division and nutritional status has also been observed in the oligochaete *Pristina leidyi* (Zattara & Bely, 2013). Our results suggest that posterior regeneration in both the presence and absence of the MPC cluster can be initiated, and new segments form in the animals with an MPC cluster. However, the compromised nutritional status of the animals in this experiment reduces the regeneration response in both groups. Our results demonstrate that the presence of the MPC cluster confers an advantage to regenerating animals, allowing them to regenerate a small number of new segments.

The results of the experiments we conducted directly demonstrate a role for cells residing within segments 4−6 in conferring regeneration ability, and provide support that the identity of these cells is within the MPC cluster. Other strategies were attempted to directly remove the MPC cluster, but proved problematic. For example, the precursor cell of the MPC cluster (3D) was deleted in cleavage stage embryos. While most larvae resulting from these deletions lack an MPC cluster, juveniles compensate for loss of the embryonic precursor cell (Dannenberg and Seaver, in preparation). Direct laser ablation (e.g., Pernet, Amiel, & Seaver, 2012; Yamaguchi & Seaver, 2013) of the MPC cluster in juveniles is not currently possible because the MPC cluster is not visible without the aid of molecular markers, prohibiting its direct observation in living animals. Clearly, further studies aided by alternative techniques are needed before the contribution of the MPC cluster to regeneration can be more thoroughly understood.

### 3.4 Importance of the nervous system in regeneration

One unexpected observation from our analyses of regeneration in individuals with and without the MPC cluster is that posterior regeneration in *C. teleta* can occur in the absence of a brain, albeit with fewer segments compared with animals that have an intact anterior end. In many annelids, the presence of a brain (supraesophageal ganglion) is required for regeneration of posterior segments (Casanova, 1955; Clark & Bonney, 1960; Clark & Clark, 1959; Durchon, 1956; Golding, 1967a; Hausenchild, 1960). The brain in these animals is proposed to secrete hormones that promote regeneration (Clark, 1959; Golding, 1967b; Herlant‐Meewis, 1964; Hofmann, 1966). However, there are also examples in which posterior regeneration can occur in the absence of the brain, such as in the two annelids *Branchiomma* and *Chaetopterus* (Hill, 1972). Notably, in annelids that can regenerate a head, the presence of brain tissue is not required for successful regeneration (see Bely, 2006). These examples highlight the substantial variation across annelids in the requirement of a brain for regeneration. In *C. teleta*, decreased posterior regeneration capability in the absence of anterior structures might simply be due to the inability of the animal to feed after amputation. However, it may also be that, although the brain is not absolutely required, it plays a role in posterior regeneration, and regeneration is impeded if the brain is removed.

Our analyses of regeneration in individuals with and without the MPC cluster suggest a functional link between the presence of dividing cells in the blastema and the extension of axons from pre‐existing segments. In specimens lacking neurite extension into the blastema, EdU‐positive cells are not detectable in the blastema. This suggests a temporal or functional link between extension of axons and the onset of cell proliferation. For example, during the initial stages of blastema formation, it is possible that a first step in the process is axon extension, and a second step is an increase in proliferating cells. Furthermore, axon extension into the blastema may be necessary for regeneration to occur. Intriguingly, in many species there is evidence suggesting that regeneration depends on the presence of neurons (reviewed in Kumar & Brockes, 2012; and including multiple studies in annelids (Berrill, 1952; Boilly‐Marer, 1971; Herlant‐Meewis, 1964; Hyman, 1940; Morgan, 1902; Müller, Berenzen, & Westheide, 2003)). Two theories explaining this phenomenon have been proposed: nerves secrete factors that activate cell proliferation and differentiation in the blastema (Herlant‐Meewis, 1964; Müller et al., 2003; Varhalmi et al., 2008), or axons act as highways to facilitate the migration of cells to the wound site (Cornec et al., 1987; Stephan‐Dubois, 1954). Based on current data, both of these alternatives are possible in *C. teleta*. Future experiments aimed at assessing the involvement of the nerve cord during *C. teleta* posterior regeneration will allow us to gain a greater understanding of the mechanisms driving a successful regenerative event.

### 3.5 Model of posterior regeneration in *C. teleta*

**Figure 7:**
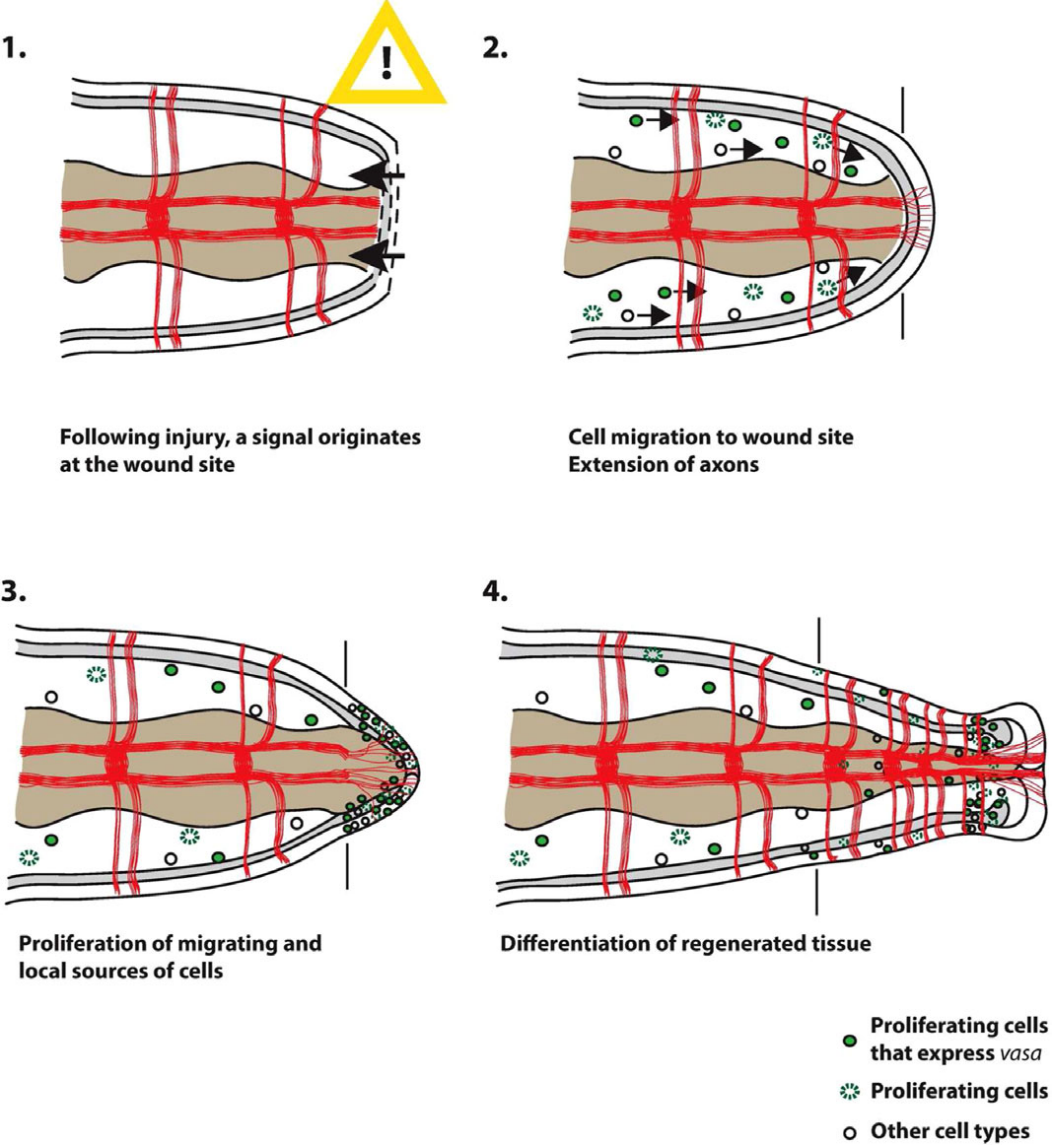
Working model of posterior regeneration in *C. teleta*. Schematic representation of the steps involved in *C. teleta* posterior regeneration. All panels show a representation of the posterior ends of amputated juveniles in ventral view, with anterior to the left. Black vertical lines indicate the amputation site in each panel. The ectoderm is shown as a white outermost layer, the mesoderm is shaded in grey, and the endoderm is shown in light brown. The coelomic cavity is represented by the space between the endoderm and mesoderm. Axons of the ventral nerve cord are shown in red. (1) Following injury, a signal originates at the wound site and stimulates a cellular response. (2) This response involves both migration of cells to the wound site and extension of axons from the pre‐existing segments into the forming blastemal tissue. A subset of cells that migrate are dividing (green), and some of these also express *CapI‐vasa* (dotted green). (3) Migrating cells reach the wound site and divide. Proliferation of cells in pre‐existing tissue close to the wound site also occurs. Both local and distant sources of cells contribute to the growing blastema. (4) Incorporation and differentiation of previously proliferating cells into the regenerated tissue

From this study and from data in other annelids, we propose a working model of posterior regeneration in *C. teleta*, which includes both local and distant sources of cells that contribute to the regenerating tissue (Fig. 7). Following injury, a signal originates at the wound site and stimulates a cellular response. This response includes the initiation of cell migration to the wound site. The origin of these migrating cells is both close to the wound site (e.g., less than one segment away) and distant from the wound site (e.g., more than one segment away). A subset of the migrating cell population are proliferating stem cells that express the stem cell marker *vasa*. A second, simultaneous response to the initial injury involves the extension of axons from the ventral nerve cord into the forming blastemal tissue. Following migration of cells to the wound site, there is at least one further round of cell division before these cells differentiate and become incorporated into a subset of tissues in the regenerated posterior segments. The remaining tissues in the regenerate are derived from proliferation of cells from local sources. In some cases, these cells may be restricted to contribute only to the germ layer from which they originate. Future studies investigating the origin and fate of cells that contribute to regenerating tissue, and the mechanistic involvement of neuronal projections, will allow us to better understand the processes that are essential for successful regeneration. This in turn may give insight into why *C. teleta* is capable of posterior, but not anterior, regeneration. Broader implications of this study include identifying aspects of annelid regeneration shared with other metazoans, and those that are unique. This will provide clues as to why some animals are highly successful regenerators, while others are not.

## 4 MATERIALS AND METHODS

### 4.1 Animal husbandry and transverse amputations

A colony of *C. teleta* was maintained in the laboratory at 16−19°C, according to published culture methods (Grassle & Grassle, 1976). Larvae were allowed to emerge naturally from the maternal brood tube, and 40 individuals were induced to metamorphosed into single glass fingerbowls containing filtered seawater (FSW) and previously frozen and sieved estuarine mud as a food source. Regeneration experiments were performed on juveniles at 2 weeks post‐metamorphosis. To isolate enough juveniles of the appropriate age for experiments, animals were initially removed from the mud and placed in 0.5% cornmeal agar:FSW plates (Sigma 42347, Darmstadt, Germany) supplemented with 60 μg/mL penicillin (Sigma P3032), plus 50 μg/mL streptomycin (Sigma S6501). Typically animals remained in agar plates for 1−4 h before amputation and fixation, which also aided in removal of mud particles from the exterior of their bodies. Prior to amputation, animals were immobilized in 0.5% cornmeal agar supplemented with MgCl_2_ (1:1 FSW:0.37 mol/L MgCl_2_), for between 15 and 30 min. Immediately before amputation, animals were placed in a drop of 0.37 mol/L MgCl_2_:FSW (1:1) on a platform of black dissecting wax (American Educational Products, Fort Collins, CO, USA) and amputated at the posterior edge of the target segment using a microsurgery scalpel (Feather; 15 degree blade, Carlsbad, CA, USA). Chaetal morphology and morphological differences between thoracic and abdominal segments allowed accurate targeting of a specific amputation position. Following amputation, animals were returned to 0.5% cornmeal agar:FSW dishes for up to 24 h. For analysis of longer periods of regeneration, animals were placed into bowls of FSW and sieved estuarine mud for the desired length of time.

### 4.2 Detection of cell proliferation by incorporation of EdU

To detect cells in S‐phase of the cell cycle, EdU (Life Technologies C10337, Carlsbad, CA, USA) was added to a dish of FSW containing whole or regenerating juveniles at a final concentration of 3 μmol/L for 1 h. Animals were then placed in 1:1 0.37 mol/L MgCl_2_:FSW for 15 min before fixation overnight at 4°C in 3.7% paraformaldehyde (PFA):FSW. Animals were then rinsed several times with phosphate‐buffered saline (PBS) and exposed to PBS + 0.5% Triton X‐100 before the EdU detection reaction was performed, following the manufacturer's recommendations. For some experiments, in situ hybridization or immunohistochemistry was performed on samples prior to detection of EdU (see appropriate sections below).

### 4.3 Cloning of *C. teleta Ct‐myc* gene

A single *C. teleta myc* homolog was identified with a tblastn search against the *C. teleta* genome (JGI; ELT88315). A recent study confirmed the presence of a single *myc* homolog in *C. teleta*, and phylogenetic analyses placed it within the Myc protein family (Bao, Xu, & Shimeld, 2017). Fragments of coding sequence were amplified by polymerase chain reaction from mixed larval stage cDNA, using gene‐specific primers (F: GAGCAACACACCCCTAATGG; R: CGAAATGACATGCTCAGAGG). A single 964 bp fragment was cloned into pGEM‐Teasy (Promega, Madison, WI, USA) and sequenced. The *C. teleta Ct‐myc* gene fragment was submitted to the National Center for Biotechnology Information as an original sequence submission with the accession number MF693912. The cloned *Ct*‐*myc* gene fragment was used as template to generate digoxigenin (DIG)‐labeled riboprobe anti‐sense RNA probe for in situ hybridization (see next section).

### 4.4 Whole‐mount in situ hybridization

Following fixation in 3.7% PFA:FSW overnight at 4°C, juveniles were washed in PBS, dehydrated through a methanol series to 100% methanol, and stored at −20°C for up to 4 weeks. A DIG‐labeled riboprobe for *CapI‐vasa* (Dill & Seaver, 2008) (BK006523) was generated with the SP6 MEGAscript kit (Ambion Inc., Austin, TX, USA) and DIG‐11‐UTP (Sigma 11209256910). The *CapI‐vasa* probe length was 1122 bp, and was diluted to a final concentration of 1 ng/μL. A DIG‐labeled riboprobe for *Ct‐myc* (MF693912) was generated with the T7 MEGAscript kit (Ambion) and DIG‐11‐UTP (Sigma). The *Ct‐myc* probe length was 964 bp, and was diluted to a final concentration of 1 ng/μL. Whole‐mount in situ hybridization was performed following published protocols (Seaver & Kaneshige, 2006). Following hybridization at 65°C for 48–72 h, the probe was detected using nitroblue tetrazolium chloride/5‐bromo‐4‐chloro‐3‐indolyl phosphate (NBT/BCIP) color substrate. Typically, the reaction was allowed to develop for 4−5 h. Extended development of the substrate reaction did not result in different expression domains for either gene. If a combination of EdU incorporation and in situ hybridization was to be performed, the in situ hybridization procedure was completed before the EdU detection reaction.

### 4.5 Immunohistochemistry

Following EdU incorporation (see Section 4.2), juveniles were washed several times in PBS + 0.1% Triton X‐100 (PBT), before being treated with block solution (PBT + 10% normal goat serum, Sigma G9023) for 45**–**60 min at room temperature. Mouse anti‐acetylated α‐tubulin antibody (Sigma T6743) was diluted 1:300 in block solution, and animals were incubated for 2−18 h at 4°C. Animals were washed twice in PBT, followed by four PBT washes of 20**–**30 min each. Donkey anti‐mouse‐546 secondary antibody (Invitrogen A21203, Carlsbad, CA, USA) was diluted 1:250 in block solution, and animals were incubated for 2**–**18 h at room temperature. Following two rinses in PBT, four PBT washes of 20**–**30 min each were conducted. Animals were imaged and analyzed as described in Section 4.8.

### 4.6 EdU pulse−amputate−wait−BrdU pulse

Two‐week‐old juveniles were exposed to EdU at a final concentration of 3 μmol/L in FSW for 1 h. Animals were then placed in 1:1 0.37 mol/L MgCl_2_:FSW for 15 min, and then amputated at the boundary between segments 10 and 11. Juveniles collected for the 0 hpa time point were then exposed to BrdU (Sigma 858811) at a final concentration of 0.1 mg/mL for 1 h, before being placed in 1:1 0.37 mol/L MgCl_2_:FSW solution for 15 min to immobilize them, and then fixed in 3.7% PFA:FSW overnight at 4°C. Regenerating juveniles were allowed to regenerate for specific time periods following amputation (1 dpa, 2 dpa, or 3 dpa), and were then collected and exposed to BrdU for 1 h, immobilized, and fixed overnight as described above. Following fixation, animals were rinsed several times with PBS and digested with 0.01 mg/mL Proteinase K (Invitrogen 25530049) in PBS for 5 min. After this incubation, the Proteinase K solution was removed and animals were re‐fixed in 3.7% PFA:PBS for 10 min. Animals were then washed three times in PBS. DNA was denatured by incubation in pre‐warmed 4 mol/L HCl for 15−30 min at 37°C. The solution was then neutralized with at least five washes of 0.1 mol/L sodium borate over 15−30 min. Animals were then washed twice in PBS, followed by exposure to PBS + 0.5% Triton X‐100, and the EdU detection reaction was performed according to the manufacturer's recommendations. Following detection of EdU, animals were placed in block solution (PBT + 10% normal goat serum; Sigma G9023) for 45 min to 1 h at room temperature. Animals were then incubated for at least 3 h at room temperature, or overnight at 4°C, in mouse anti‐BrdU antibody (1:250; Thermo Fisher MoBU1, B35128, Carlsbad, CA, USA) and rabbit anti‐acetylated α‐tubulin antibody (1:500; Cell Signaling 5335T, Danvers, MA, USA), diluted in block solution. Following incubation, primary antibody was removed by two rinses in PBT, followed by four PBT washes of 20−30 min each. Animals were then incubated in donkey anti‐mouse‐546 secondary antibody (1:250; Invitrogen A21203) and goat anti‐rabbit‐647 secondary antibody (1:250; Invitrogen A21245), diluted in block solution, for 2−4 h at room temperature. Following secondary antibody incubation, samples were rinsed twice in PBT, followed by four PBT washes of 20−30 min each.

### 4.7 Statistical analyses

*CapI‐vasa*‐expressing cells in the MPC cluster were manually counted at 0 hpa, 1 dpa, 2 dpa, and 3 dpa using a compound microscope. Cell counts were taken from at least 55 individuals for each time point, and comparisons were made between the number of *CapI‐vasa*‐expressing cells at 0 hpa and either 1 dpa, 2 dpa, or 3 dpa. Statistical significance was determined using a Student's two‐tailed *t* test. Differences in the number of *CapI‐vasa*‐expressing cells in the MPC cluster were considered statistically significant if the *P* value was <0.05. The proportion of juveniles with *CapI‐vasa*‐expressing cells in their coelomic cavity at 0 hpa was compared with the proportion of juveniles that contained *CapI‐vasa‐*expressing cells in their coelomic cavity at 1 dpa, 2 dpa, or 3 dpa. Juveniles were counted as possessing *CapI‐vasa*‐expressing cells in their coelomic cavity if any cells were seen in any segment, regardless of the number of cells. At least 63 individuals for each time point were included. Statistical significance was determined using a two‐tailed *Ζ* test. A *P* value <0.05 was considered statistically significant.

### 4.8 Microscopy and imaging

Before imaging, animals were placed in 80% glycerol:PBS plus 0.125 μg/μL Hoechst 33342 (Life Technologies H3570) overnight. Specimens analyzed by in situ hybridization alone were imaged using an Axioskop 2 motplus compound microscope (Zeiss, Gottingen, Germany), coupled with a SPOT FLEX digital camera (Diagnostic Instruments Inc., Sterling Heights, MI, USA). Images were captured using SPOT imaging software (version 5.2). All other specimens containing a combination of EdU, BrdU, and/or immunohistochemistry and in situ hybridization were imaged using a Zeiss LSM 710 confocal microscope (Zeiss, Gottingen, Germany). *Z*‐stack projections were generated using Fiji (Schindelin et al., 2012). All figures were created in Adobe Photoshop CS6 (version 13.0) or Adobe Illustrator CS6 (version 16.0.0).

## CONFLICT OF INTEREST

The authors have no conflicts of interest to declare.

## Supporting information

Figure S1. Proliferating cells are present in all three germ layers during posterior regeneration. All panels show confocal stacks of posterior ends of amputated juveniles in ventral view, with anterior to the left. The panels in each row are from a single individual. The time following amputation is listed to the left of rows, and the antibody/chemical represented in each panel is listed at the top of columns. White vertical lines indicate the approximate position of the amputation site. Amputations were conducted at the boundary of segments 10 and 11. (A), (B) Anti‐acetylated α‐tubulin reactivity (red) and EdU incorporation (green), combined with a transmitted light image of the regenerated tissue (Ac‐tub + EdU + TL). (A′), (B′) EdU incorporation (green), combined with a transmitted light image of the regenerated tissue (EdU + TL). In (A′) and (B′), white arrows denote EdU‐positive cells in the ectoderm, white arrowheads show EdU‐positive cells in the mesoderm, and black arrows point to EdU‐positive cells in the endoderm. Ac‐tub, anti‐acetylated tubulin; TL, transmitted light. Scale bars in all panels represent 50 μm.

Figure S2. Stable position of the MPC cluster and distribution of *CapI‐vasa*‐positive coelomic cavity cells in juveniles. All juveniles were amputated between segments 10 and 11. Segment number is indicated at the top of columns 1–10, with each row representing an individual juvenile. The time following amputation when animals were sampled is listed to the left of rows. Green shading indicates the segments where the MPC cluster was located, as visualized by *CapI‐vasa* expression. Black shading indicates segments where *CapI‐vasa*‐positive coelomic cavity cells were seen. A black and green dotted line indicates segments with both the MPC cluster and *CapI‐vasa*‐positive cells in the coelomic cavity. Multiple ×s mean the tenth segment was missing in that particular juvenile.
