# Supplementary figures and images for: Investigation into the cellular origins of posterior regeneration in the annelid Capitella teleta
Source: Regeneration (Oxf). 2017 Dec 6;5(1):61–77. doi: 10.1002/reg2.94 (PMC5911572; doi:10.1002/reg2.94)

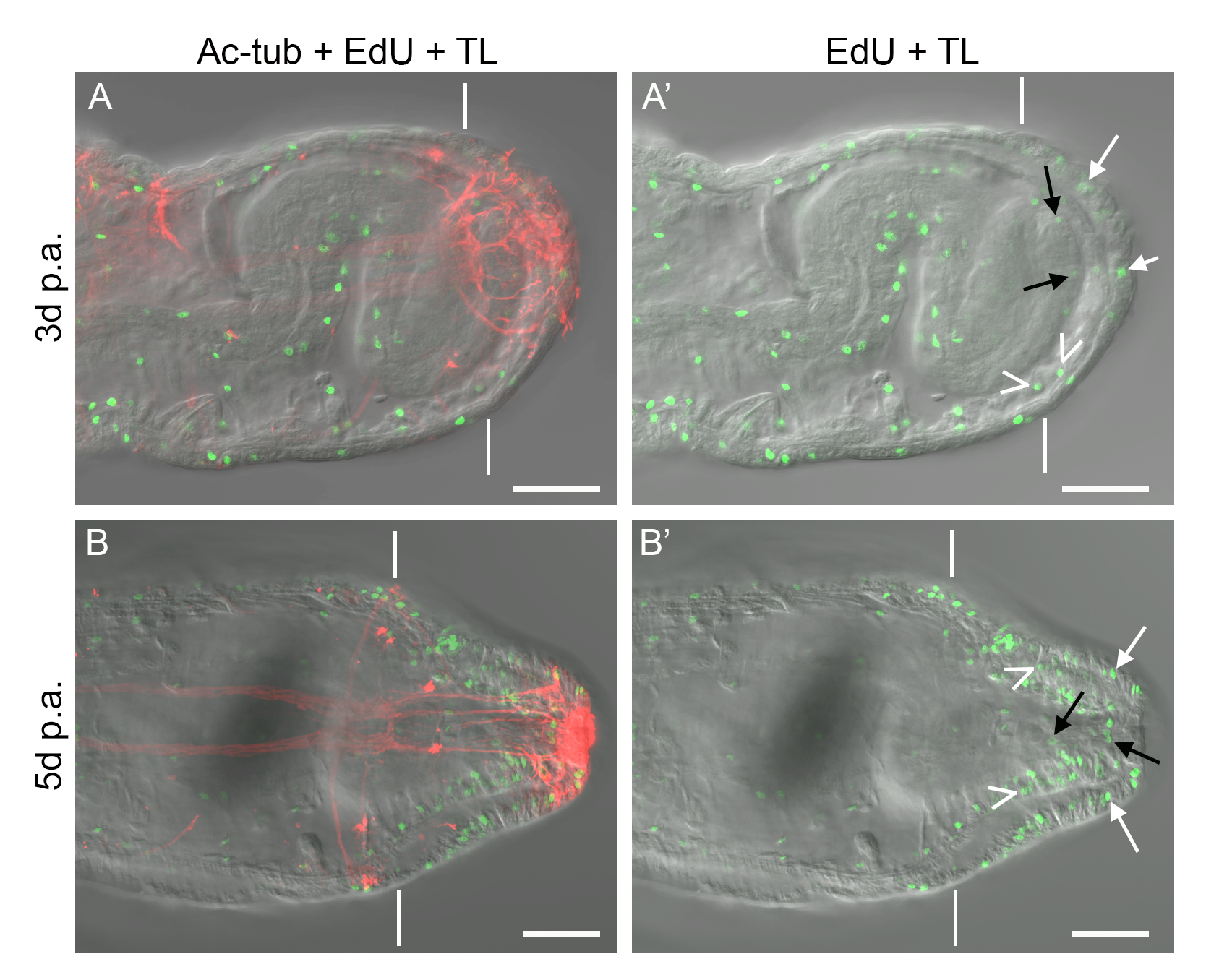

Supplement: Supplementary file 1 — Figure S1. Proliferating cells are present in all three germ layers during posterior regeneration. All panels show confocal stacks of posterior ends of amputated juveniles in ventral view, with anterior to the left. The panels in each row are from a single individual. The time following amputation is listed to the left of rows, and the antibody/chemical represented in each panel is listed at the top of columns. White vertical lines indicate the approximate position of the amputation site. Amputations were conducted at the boundary of segments 10 and 11. (A), (B) Anti‐acetylated α‐tubulin reactivity (red) and EdU incorporation (green), combined with a transmitted light image of the regenerated tissue (Ac‐tub + EdU + TL). (A′), (B′) EdU incorporation (green), combined with a transmitted light image of the regenerated tissue (EdU + TL). In (A′) and (B′), white arrows denote EdU‐positive cells in the ectoderm, white arrowheads show EdU‐positive cells in the mesoderm, and black arrows point to EdU‐positive cells in the endoderm. Ac‐tub, anti‐acetylated tubulin; TL, transmitted light. Scale bars in all panels represent 50 μm. [file REG2-5-61-s001.tif]

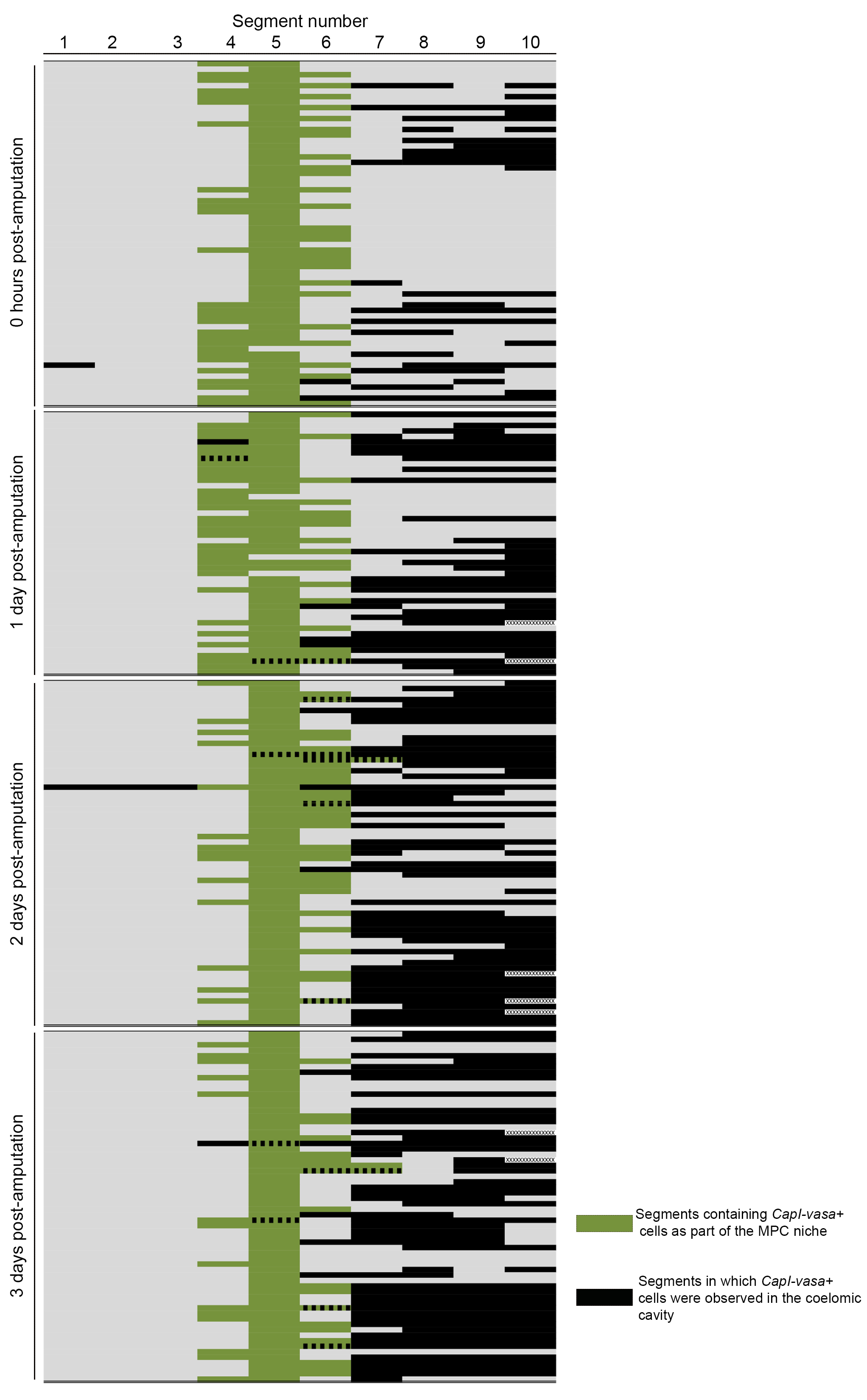

Supplement: Supplementary file 2 — Figure S2. Stable position of the MPC cluster and distribution of CapI‐vasa‐positive coelomic cavity cells in juveniles. All juveniles were amputated between segments 10 and 11. Segment number is indicated at the top of columns 1–10, with each row representing an individual juvenile. The time following amputation when animals were sampled is listed to the left of rows. Green shading indicates the segments where the MPC cluster was located, as visualized by CapI‐vasa expression. Black shading indicates segments where CapI‐vasa‐positive coelomic cavity cells were seen. A black and green dotted line indicates segments with both the MPC cluster and CapI‐vasa‐positive cells in the coelomic cavity. Multiple ×s mean the tenth segment was missing in that particular juvenile. [file REG2-5-61-s002.tif]
